# Associations of Serum n–6 Polyunsaturated Fatty Acid Concentrations with Heart Rate-Corrected QT and JT Intervals in Middle-Aged Males

**DOI:** 10.1016/j.tjnut.2025.09.012

**Published:** 2025-09-25

**Authors:** Haleh Esmaili, Behnam Tajik, Tomi-Pekka Tuomainen, Jussi Kauhanen, Sudhir Kurl, Jukka T Salonen, Jyrki K Virtanen

**Affiliations:** 1 Institute of Public Health and Clinical Nutrition, University of Eastern Finland, Kuopio Campus, Kuopio, Finland; 2 Department of Health Sciences, University of York, York, United Kingdom; 3 Department of Public Health, Faculty of Medicine, University of Helsinki, Helsinki, Finland; 4 MAS-Metabolic Analytical Services Oy, Helsinki, Finland

**Keywords:** n–6 polyunsaturated fatty acids, QT interval, JT interval, population study, arrhythmias

## Abstract

**Background:**

Although n–6 (ω-6) polyunsaturated fatty acids (PUFAs), particularly the main n–6 PUFA linoleic acid (LA), have been shown to be inversely related to cardiovascular disease (CVD) risk, their associations with ventricular repolarization, a marker of ventricular arrhythmias, remain unclear.

**Objectives:**

This study examined the relationships between serum n–6 PUFA concentrations and heart rate-corrected QT (QTc) and JT (JTc) intervals.

**Methods:**

This cross-sectional study included 1420 Finnish males (aged 42–60 y) free of CVD from the Kuopio Ischaemic Heart Disease Risk Factor Study. Fasting serum n–6 PUFA concentrations were determined by gas chromatography, and electrocardiographic intervals were automatically derived. QTc and JTc intervals were calculated using Bazett’s formula. Associations were analyzed using multivariable-adjusted analysis of covariance.

**Results:**

Higher serum total n–6 PUFA concentrations were associated with shorter QTc and JTc intervals [QTc, extreme-quartile difference: −3.36 ms; 95% confidence interval (CI): −6.69, −0.41; *P*-trend = 0.02; JTc, extreme-quartile difference: −3.38 ms; 95% CI: −6.79, −0.27; *P*-trend = 0.03]. Similar inverse associations were found for serum LA (QTc: −4.64 ms; 95% CI: −8.00, −1.27; *P*-trend = 0.009; JTc: −3.99 ms; 95% CI: −7.44, −0.54; *P*-trend = 0.02). The other n–6 PUFAs—γ-linolenic acid, dihomo-γ-linolenic acid, and arachidonic acid—showed no significant associations.

**Conclusions:**

Higher serum total n–6 PUFA and LA concentrations are associated with shorter QTc and JTc intervals, suggesting a potential protective effect on ventricular arrhythmias.

## Introduction

Cardiovascular diseases (CVDs) are the leading cause of death globally [1]. Despite the potential proinflammatory characteristics of the n–6 (ω-6) PUFAs, recent evidence has rather depicted that n–6 PUFAs, particularly linoleic acid (LA, 18:2n–6), are associated with a lower risk of CVD, CVD-related mortality, and sudden cardiac death [[2], [3], [4]]. LA, the main n–6 PUFA, is primarily found in vegetable oils, seeds, and nuts [4,5]. In the body, LA acts as a precursor to longer-chain unsaturated n–6 PUFAs, including γ-linolenic acid (GLA, 18:3n–6), dihomo-γ-linolenic acid (DGLA, 20:3n–6), and arachidonic acid (AA, 20:4n–6), through the desaturase and elongase enzymes [5,6]. In addition, AA can be found in certain foods, such as meats and eggs [5].

The electrocardiogram (ECG) is a simple and noninvasive diagnostic method widely used for cardiac function abnormalities [7]. QT and JT intervals are common ECG indices that can be applied in the prevention and diagnosis of ventricular arrhythmia, reflecting durations of myocardial repolarization and ventricular abnormality [[8], [9], [10]]. Because the QT interval includes both depolarization and repolarization, it has been suggested that the JT interval, which is only related to repolarization, can be a more appropriate measure of ventricular repolarization, especially when QT prolongation relates to higher QRS complex duration [8,9]. Furthermore, heart rate-corrected QT (QTc) and JT (JTc) intervals are associated with future CVD events [9,10]. Abnormal QT prolongation is related to left ventricular hypertrophy, cardiac arrhythmia, ventricular tachycardia, and torsade de pointes [11]. According to a meta-analysis, a prolonged QT and JT interval is a predictor of mortality and has been associated with a higher risk of CVD and sudden cardiac death [12,13].

Despite the established beneficial association of LA with CVD, particularly through total and LDL cholesterol reductions [2], limited knowledge exists about the relationship between n–6 PUFAs and ventricular repolarization (QT and JT intervals), indicators of arrhythmia risk [14,15]. Therefore, our study aimed to assess the relationship between the serum n–6 PUFA concentrations and QTc and JTc intervals among middle-aged and older males from the Kuopio Ischaemic Heart Disease Risk Factor Study (KIHD).

## Methods

### Study population

In the current cross-sectional study, we used data from the KIHD cohort, collected at the baseline examinations of the KIHD between 1984 and 1989. The KIHD is a prospective population-based study to investigate risk factors for CVD, carotid atherosclerosis, and related outcomes. The participants are an age-stratified sample of males from eastern Finland [16]. At the baseline, 2682 males (82.9% of the eligible) aged 42, 48, 54, or 60 y took part in the examinations. The KIHD study protocol was approved by the research ethics committee of the University of Kuopio (approval no. 01/12/1983). All participants provided written informed consent.

From the analyses, we excluded participants with a history of CVD, with missing data on serum n–6 PUFAs or on QT and JT intervals, or a QRS complex of ≥120 ms because a higher QRS complex affects the QT interval [9]. After the exclusions, 1420 males were included in the final analysis (Supplemental Figure 1).

### Measurements

Fasting venous blood samples were collected at the baseline examination between 08:00 and 10:00. Subjects were instructed to abstain from ingesting alcohol for 3 d, smoking for 12 h, and eating for 12 h. Details of the serum lipid and lipoprotein measurements, medical history and medication assessment, smoking, and alcohol consumption have been reported previously [17]. An immunometric assay (Immulite High Sensitivity C-reactive Protein Assay; DPC) was used to measure serum high-sensitivity C-reactive protein (CRP). Physical activity (kilocalories per day) was evaluated according to the 12-mo leisure-time physical activity questionnaire and included the average duration, intensity, and frequency of each activity [18]. Hypertension was defined as systolic/diastolic blood pressure of >140/90 mm Hg, clinical diagnosis of hypertension, or use of hypertension medication [19]. Height and weight were measured by a study nurse during the study visit. BMI was computed as the ratio of weight in kilograms to the square of height in meters.

The food intakes were assessed at the time of blood sampling with an instructed 4-d food diary (3 weekdays and 1 weekend) by household measures [20]. The completed food records were checked within a week of filling them by a dietician at the study visit when the participants also gave blood samples for, e.g., measurements of the serum fatty acid concentrations. The information on education and annual income was obtained by using self-administered questionnaires.

### Serum fatty acid measurements

Esterified and nonesterified serum fatty acids were measured from stored samples that had been frozen at −80°C in 1 gas chromatographic run without preseparation as described previously [21]. Serum fatty acids were extracted with chloroform and methanol. The chloroform phase was evaporated and treated with sodium methoxide, which methylated and esterified fatty acids. Quantification was carried out with reference standards (Check Prep Inc.). Each analyte had an individual reference standard, and the internal standard was eicosane. Chromatographic determination of fatty acids was done in an NB-351 capillary column (HNU-Nordion) using a Hewlett-Packard 5890 Series II gas chromatograph (Hewlett-Packard Company; since 1999 Agilent Technologies Inc.) with a flame ionization detector. Results were obtained in micromoles per liter, and in the data analyses, the proportion of a fatty acid in the serum total fatty acids was used.

The coefficient of variation (CV%) was 8.7% for LA, 11.6% for GLA, 8.3% for DGLA, and 9.9% for AA. For the serum total n–6 PUFA concentration, we used the sum of LA, GLA, DGLA, and AA. For the serum n–3 PUFAs that were used as covariates, the CV% were 8.6% for α-linolenic acid (ALA; 18:3n–3), 9.4% for EPA (20:5n–3), 12.7% for docosapentaenoic acid (DPA; 22:5n–3), and 11.9% for DHA (22:6n–3). The serum long-chain n–3 PUFA concentration was the sum of serum EPA, DPA, and DHA, and the serum total n–3 PUFA concentration was the sum of serum ALA, EPA, DPA, and DHA.

### Assessment of electrocardiogram

All electrocardiographic intervals and amplitude were recorded automatically at the baseline examinations using a standard 12-lead electrocardiogram (ECG) with a paper speed of 50 mm/s [22]. The QT interval was measured from the onset of the QRS complex to the end of the T wave; the last was characterized as the intersection of the isoelectric line and the tangent of the maximal slope on the downward limb of the T wave [23]. Because there is a strong correlation between the QT interval and heart rate, the Bazett’s formula was used for calculating the QTc and JTc intervals [24] as follows: QTc = QT/√RR (RR = the interval between 2 consecutive R waves in the ECG); and JTc = QTc−QRS.

### Statistical analysis

The associations between the serum total n–6 PUFA concentration and demographic, lifestyle, and clinical characteristics were assessed by means and linear regression for continuous variables and chi-square test for categorical variables. Correlations between the serum total and serum individual n–6 PUFA concentrations were evaluated by Spearman correlation coefficients.

The mean values of QTc and JTc intervals in the quartiles of the n–6 PUFA were analyzed using analysis of covariance, adjusted for potential confounding factors. Three different models were used to control for potential confounding factors. The covariables in the analyses were selected based on the previous studies in the KIHD with the serum n–3 PUFAs [25] or the associations with exposures or outcomes in the present analysis.

Model 1 was adjusted for age (years) and examination year. Model 2 included the variables in model 1 plus leisure-time physical activity (kilocalories per day), smoking status (never smoker, previous smoker, current smoker <20 cigarettes/d, and current smoker ≥20 cigarettes/d), serum total long-chain n–3 PUFA concentration (sum of DHA, DPA, and EPA as a percentage of serum total fatty acids), alcohol consumption (grams per week), BMI, hypertension (yes/no), and type 2 diabetes (yes/no). Model 3 included the variables in model 2 plus potential effect mediators serum LDL and HDL cholesterol concentration (millimoles per liter), serum triglyceride concentration (millimoles per liter), and serum high-sensitive CRP concentration (milligrams per liter). We also tried adjusting model 2 further for dietary factors including energy intake, saturated fatty acid intake, fiber intake, and vegetable, fruit, and berry intake. However, as these adjustments did not appreciably change the associations (<5% change in the estimates), they were not included in the final models. Cohort means of covariates were used to replace missing values (<0.5% of the values). Testing of the linear trend across the n–6 PUFA quartiles was conducted by assigning the median values for each fatty acid quartile and using those as a single continuous variable.

To examine whether the associations between the serum n–6 PUFAs and QTc and JTc intervals were influenced by potential effect modifiers, we conducted interaction analyses of age, BMI, leisure-time physical activity, alcohol intake, and serum concentrations of long-chain n–3 PUFAs and total n–3 PUFA. In these analyses, the statistical significance of the interactions on a multiplicative scale was assessed by stratified analysis and likelihood ratio tests using a cross-product term. Interaction terms were created by multiplying the standardized scores (*z*-scores) of the median of each n–6 PUFA with each potential modifier, and these terms were included in the multivariable-adjusted models (model 2), alongside the main effects of the serum n–6 PUFAs and the effect modifiers. Restricted cubic spline analysis was used to evaluate the associations of dietary LA and AA intakes with their serum concentrations. All *P* values were 2-sided (*α* ≤ 0.05). Data were analyzed using SPSS software version 27 for Windows (IBM Corp.) and Stata version 14 for spline analysis (Stata Corp).

## Results

### Study population characteristics

The mean ± SD age of the participants was 52.0 ± 5.4 y. The mean ± SD serum n–6 PUFA concentrations, as a percentage of all serum fatty acids, were 33.11 ± 4.62 for total n–6 PUFA, 26.68 ± 4.44 for LA, 0.29 ± 0.11 for GLA, 1.34 ± 0.27 for DGLA, and 4.81 ± 1.01 for AA.

The intercorrelations between the individual n–6 PUFAs were weak, except for GLA and DGLA (*r* = 0.56). The other correlation coefficients (*r*) were −0.21 for LA and GLA, −0.09 for LA and DGLA, 0.11 for LA and AA, 0.16 for AA and GLA, and 0.09 for AA and DGLA.

Serum LA concentration had a nonlinear association with the estimated dietary intake of LA (Supplemental Figure 2). The increase in serum LA concentration was relatively linear with LA intake up to ∼4% of energy, after which the increase was smaller (*P*-nonlinearity < 0.001) (Supplemental Figure 2). In addition, the estimated dietary AA intake had a positive association with the serum AA concentration (Supplemental Figure 2). We did not have information on the intakes of GLA and DGLA.

The characteristics of the participants according to quartiles of the serum total n–6 PUFA concentration are presented in Table 1. Males with higher serum total n–6 PUFA concentrations were younger, were more educated, and had higher physical activity levels and lower smoking rates. They were less likely to have diabetes and hypertension. Moreover, they had lower consumption of alcohol and saturated fatty acids, a lower BMI, lower CRP concentrations, lower serum triglyceride concentrations, and higher HDL cholesterol concentrations.

**TABLE 1:** Population characteristics according to the quartiles of total n–6 PUFA concentration^1^.

Variables	Serum total n–6 PUFA quartile (% of all serum fatty acids)				*P*-trend^2^
	Q1 (<30.06)(*n* = 355)	Q2 (30.06–33.36)(*n* = 355)	Q3 (33.37–36.13)(*n* = 355)	Q4 (>36.13)(*n* = 355)	
Age (y)	52.7 ± 5.0	52.3 ± 5.2	51.9 ± 5.4	51.0 ± 5.9	<0.001
Educations (y)	8.7 ± 3.4	8.7 ± 3.4	9.2 ± 3.8	9.7 ± 3.8	<0.001
Income (euro)	13,148 ± 8435	14,015 ± 9842	14,553 ± 9474	15,372 ± 9659	0.001
BMI (kg/m^2^)	28.21 ± 4.00	27.18 ± 3.42	26.08 ± 2.83	25.49 ± 2.78	<0.001
Physical activity (kcal/d)	119 ± 159	133 ± 159	139 ± 154	163 ± 205	0.001
Current smoker (%)	36.9	31.0	30.4	23.9	0.003
Alcohol consumption (g/wk)	99 ± 149	72 ± 113	63 ± 96	53 ± 83	<0.001
Diabetes (%)	6.8	5.1	3.1	2.8	0.036
Hypertension (%)	67.9	57.5	49.0	47.6	<0.001
Medication use (%)^3^	19.7	12.4	10.7	9.0	<0.001
Dietary SFAs (E%)	18.4 ± 4.1	18.8 ± 3.9	18.4 ± 3.5	17.6 ± 3.4	<0.001
Dietary carbohydrates (E%)	41.6 ± 6.5	41.9 ± 6.2	41.6 ± 6.2	42.4 ± 5.7	0.110
Energy intake (kcal/d)	2445 ± 600	2523 ± 679	2463 ± 578	2521 ± 619	0.258
C-reactive protein (mg/L)	2.98 ± 6.48	1.97 ± 3.70	1.92 ± 2.88	1.66 ± 2.85	<0.001
Blood glucose (mmol/L)	4.97 ± 1.40	4.74 ± 0.82	4.62 ± 0.73	4.53 ± 0.82	<0.001
Serum triglycerides (mmol/L)	1.85 ± 1.07	1.24 ± 0.52	1.03 ± 0.43	0.88 ± 0.35	<0.001
Serum HDL cholesterol (mmol/L)	1.18 ± 0.26	1.28 ± 0.29	1.33 ± 0.29	1.37 ± 0.29	<0.001
Serum LDL cholesterol (mmol/L)	3.94 ± 0.99	4.09 ± 0.98	4.06 ± 0.95	4.00 ± 1.02	0.479
Serum total cholesterol (mmol/L)	5.94 ± 1.06	5.93 ± 1.02	5.86 ± 1.00	5.79 ± 1.08	0.038
Systolic blood pressure (mmHg)	138 ± 17	136 ± 16	131 ± 15	132 ± 16	<0.001
Diastolic blood pressure (mmHg)	92 ± 11	90 ± 10	88 ± 10	88 ± 11	<0.001
Serum total long-chain n–3 PUFA (%)^4^^,^^5^	4.80 ± 2.07	4.76 ± 1.59	4.79 ± 1.48	4.72 ± 1.15	0.013
Serum LA (%)	21.19 ± 2.41	25.36 ± 1.38	28.08 ± 1.33	32.07 ± 2.52	<0.001
Serum GLA (%)	0.31 ± 0.12	0.28 ± 0.10	0.28 ± 0.10	0.27 ± 0.11	<0.001
Serum DGLA (%)	1.33 ± 0.27	1.36 ± 0.28	1.34 ± 0.28	1.33 ± 0.26	0.544
Serum AA (%)	4.32 ± 1.00	4.80 ± 0.98	4.99 ± 0.93	5.13 ± 0.94	<0.001

Abbreviations: AA, arachidonic acid; DGLA, dihomo-γ-linolenic acid; E%, percent of energy intake; GLA, γ-linolenic acid; LA, linoleic acid; Q, quartile.

1 Values are means ± SD or percentages. Data were analyzed with analysis of variance and linear regression for continuous variables and χ^2^ test for categorical variables.

2 *P*-trend refers to the linear trend across the fatty acid quartiles.

3 Antihypertensive and antihyperlipidemia medications.

4 Proportion of all serum fatty acids.

5 Serum total long-chain n–3 PUFA was the sum of DHA, docosapentaenoic acid, and EPA.

### Association of the serum n–6 PUFA concentrations with QTc and JTc intervals

The mean ± SD QTc and JTc intervals were 416.0 ± 21.8 ms and 314.0 ± 22.3 ms, respectively. After multivariable adjustments, higher serum total n–6 PUFA concentration was associated with lower QTc (the mean difference between extreme quartiles: −3.36 ms; 95% CI: −6.69, −0.41; *P*-trend across quartiles = 0.02) (Table 2, model 2). An association similar to that with serum total n-6 PUFA was also observed with LA (the mean difference between extreme quartiles: −4.64 ms; 95% CI: −8.00, −1.27; *P*-trend across quartiles = 0.009) (Table 2, model 2). Adjusting the model 2 further for the potential mediators of the associations slightly strengthened the associations (Table 2, model 3). No significant associations were found between serum GLA, DGLA, or AA and QTc (Table 2).

**TABLE 2:** Mean QTc intervals in quartiles of serum n–6 PUFAs.

Serum n–6 PUFAs	Exposure quartile				*P*-trend^1^	Mean difference between the highest and the lowest quartile (95% CI)
	1 (*n* = 355)	2 (*n* = 355)	3 (*n* = 355)	4 (*n* = 355)		
Total n–6 PUFAs (%)	<30.06	30.06–33.36	33.37–36.13	>36.13		
Model 1	420.2 (418.0, 422.5)^2^	416.0 (413.7, 418.2)	413.1 (410.8, 415.3)	414.7 (412.5, 417.0)	<0.001	−5.48 (−8.67, −2.29)
Model 2	418.9 (416.6, 421.1)	415.8 (413.6, 418.0)	413.8 (411.6, 416.0)	415.3 (413.2, 417.8)	0.02	−3.36 (−6.69, −0.41)
Model 3	419.7 (417.1, 422.2)	415.9 (413.7, 418.1)	413.5 (411.3, 415.7)	414.8 (412.4, 417.2)	0.008	−4.86 (−8.68, −1.04)
LA (%)	<23.75	23.75–26.76	26.77–29.53	>29.53		
Model 1	420. 4 (418.2, 422.7)	415.1 (412.8, 417.3)	414.5 (412.2, 416.7)	414.0 (411.8, 416.3)	<0.001	−6.39 (−9.58, −3.20)
Model 2	419.2 (416.9, 421.6)	415.3 (413.1, 417.5)	414.8 (412.6, 417.0)	414.6 (412.3, 416.9)	0.009	−4.64 (−8.00, −1.27)
Model 3	419.9 (417.4, 422.4)	415.3 (413.1, 417.5)	414.7 (412.4, 416.9)	414.1 (411.7, 416.5)	0.005	−5.76 (−9.49, −2.02)
GLA (%)	<0.21	0.21–0.27	0.28–0.35	>0.35		
Model 1	414.5 (412.3, 416.8)	416.3 (414.1, 418.6)	415.6 (413.3, 417.9)	417.5 (415.2, 419.8)	0.11	2.96 (−0.24, 6.17)
Model 2	414.7 (412.5, 416.9)	416.6 (414.3, 418.8)	415.3 (413.1, 417.5)	417.4 (415.2, 419.6)	0.16	2.73 (−0.44, 5.90)
Model 3	414.6 (412.4, 416.8)	416.5 (414.3, 418.7)	415.4 (413.2, 417.6)	417.5 (415.2, 419.7)	0.13	2.87 (−0.32, 6.05)
DGLA (%)	<1.15	1.15–1.33	1.34–1.51	>1.51		
Model 1	413.6 (411.4, 415.9)	417.3 (415.0, 419.5)	415.7 (413.4, 417.9)	417.4 (415.1, 419.6)	0.05	3.72 (0.53, 6.91)
Model 2	414.0 (411.7, 416.2)	417.2 (415.0, 419.4)	415.6 (413.4, 417.8)	417.1 (414.9, 419.4)	0.11	3.23 (−0.02, 6.48)
Model 3	414.1 (411.8, 416.3)	417.3 (415.1, 419.5)	415.5 (413.3, 417.7)	417.1 (414.8, 419.3)	0.15	3.01 (−0.29, 6.30)
AA (%)	<4.13	4.13–4.75	4.76–5.44	>5.44		
Model 1	416.5 (414.2, 418.8)	415.8 (413.6, 418.1)	413.8 (411.6, 416.1)	417.8 (415.5, 420.0)	0.64	1.30 (−1.90, 4.50)
Model 2	415.0 (412.7, 417.4)	415.6 (413.4, 417.9)	414.6 (412.4, 416.8)	418.7 (416.3, 421.0)	0.07	3.64 (−0.18, 7.11)
Model 3	414.9 (412.4, 417.4)	415.9 (413.7, 418.1)	414.5 (312.3, 416.8)	418.6 (416.2, 421.1)	0.09	3.73 (−0.07, 7.53)

Model 1: adjusted for age and examination years. Model 2: adjusted for model 1 plus smoking status, leisure-time physical activity, alcohol intake, BMI, hypertension, type 2 diabetes, and serum long-chain n–3 PUFA concentrations. Model 3: adjusted for model 2 plus serum concentrations of LDL and HDL cholesterol and triglycerides and high-sensitivity C-reactive protein.

Abbreviations: AA, arachidonic acid; CI, confidence interval; DGLA, dihomo-γ-linolenic acid; GLA, γ-linolenic acid; LA, linoleic acid; QTc, heart rate-corrected QT interval.

1 *P*-trend refers to the linear trend across the fatty acid quartiles.

2 Values are means (95% CI), analyzed with analysis of covariance.

Regarding JTc, higher serum total n–6 PUFA and LA concentrations were also associated with lower JTc (for total n–6 PUFA, the mean difference between extreme quartiles: −3.38 ms; 95% CI: −6.79 to −0.27, *P*-trend across quartiles = 0.03; for LA, the mean difference between extreme quartiles: −3.99 ms; 95% CI −7.44 to −0.54; *P*-trend across quartiles = 0.02) (Table 3, model 2). As with QTc, the associations were slightly stronger after adjustment for potential mediators of the associations (Table 3, model 3).

**TABLE 3:** Mean JTc interval in quartiles of serum n–6 PUFAs.

Serum n–6 PUFAs	Exposure quartile				*P*-trend^1^	Mean difference between the highest and the lowest quartile (95% CI)
	1 (*n* = 355)	2 (*n* = 355)	3 (*n* = 355)	4 (*n* = 355)		
Total n–6 PUFAs (%)	<30.06	30.06–33.36	33.37–36.13	>36.13		
Model 1	318.1 (315.8, 320.4)^2^	314.1 (311.8, 316.4)	311.3 (309.1, 313.6)	312.6 (310.3, 314.9)	<0.001	−5.51 (−8.78, −2.24)
Model 2	316.8 (314.4, 319.1)	313.9 (311.7, 316.2)	312.1 (309.8, 314.4)	313.4 (311.1, 315.7)	0.03	−3.38 (−6.79, −0.27)
Model 3	317.1 (314.5, 319.7)	314.0 (311.8, 316.3)	311.9 (310.6, 315.5)	313.1 (310.6, 315.5)	0.03	−4.05 (−7.97, −0.13)
LA (%)	<23.75	23.75–26.76	26.77–29.53	>29.53		
Model 1	318.1 (315.8, 320.4)	313.4 (311.1, 315.7)	312.4 (310.1, 314.7)	312.3 (310.0, 314.6)	<0.001	−5.75 (−9.02, −2.48)
Model 2	316.9 (314.5, 319.3)	313.6 (311.4, 315.9)	312.7 (310.5, 315.0)	312.9 (310.6, 315.3)	0.02	−3.99 (−7.44, −0.54)
Model 3	317.1 (314.5, 319.6)	313.7 (311.4, 315.9)	312.7 (310.4, 315.0)	312.7 (310.3, 315.2)	0.03	−4.36 (−8.20, −0.52)
GLA (%)	<0.21	0.21–0.27	0.28–0.35	>0.35		
Model 1	311.8 (309.5, 314.1)	315.0 (312.7, 317.3)	313.9 (311.6, 316.2)	315.0 (313.1, 317.7)	0.25	3.62 (0.35, 6.89)
Model 2	313.3 (311.0, 315.6)	314.4 (312.2, 316.7)	313.4 (311.2, 315.7)	315.0 (312.8, 317.3)	0.39	1.76 (−1.49, 5.01)
Model 3	313.2 (310.9, 315.5)	314.4 (312.1, 316.6)	313.5 (311.2, 315.7)	315.2 (312.9, 317.4)	0.32	1.97 (−1.30, 5.23)
DGLA (%)	<1.15	1.15–1.33	1.34–1.51	>1.51		
Model 1	311.8 (309.5, 314.1)	315.0 (312.7, 317.3)	313.9 (311.6, 316.2)	315.4 (313.1, 317.7)	0.06	3.62 (0.35, 6.89)
Model 2	312.2 (309.9, 314.5)	314.9 (312.7, 317.2)	313.9 (311.6, 316.1)	315.2 (312.9, 317.5)	0.13	2.99 (−0.34, 6.33)
Model 3	312.2 (309.9, 314.6)	315.0 (312.7, 317.2)	313.8 (311.5, 316.1)	315.2 (312.8, 317.5)	0.15	2.91 (−0.47, 6.29)
AA (%)	<4.13	4.13–4.75	4.76–5.44	>5.44		
Model 1	315.1 (312.8, 317.4)	313.6 (311.3, 315.9)	311.7 (309.4, 314.1)	315.7 (313.4, 318.1)	0.90	0.65 (−2.62, 3.93)
Model 2	313.5 (311.1, 315.9)	313.3 (311.1, 315.6)	312.6 (310.3, 314.8)	316.8 (314.4, 319.1)	0.11	3.28 (−0.27, 6.82)
Model 3	313.1 (310.5, 315.6)	313.5 (311.2, 315.8)	312.6 (310.4, 314.9)	317.0 (314.5, 319.5)	0.06	3.93 (0.03, 7.82)

Model 1: adjusted for age and examination years. Model 2: adjusted for model 1 plus smoking status, leisure-time physical activity, alcohol intake, BMI, hypertension, type 2 diabetes, and serum long-chain n–3 PUFA concentrations. Model 3: adjusted for model 2 plus serum concentrations of LDL and HDL cholesterol and triglycerides and high-sensitivity C-reactive protein.

Abbreviations: AA, arachidonic acid; CI, confidence interval; DGLA, dihomo-γ-linolenic acid; GLA, γ-linolenic acid; JTc, heart rate-corrected JT interval; LA, linoleic acid.

1 *P*-trend refers to the linear trend across the fatty acid quartiles.

2 Values are means (95% CI), analyzed with analysis of covariance.

Serum GLA, DGLA, and AA concentrations were not associated with JTc. Age, BMI, physical activity, alcohol consumption, the serum long-chain n–3 PUFA concentration, or the serum total n–3 PUFA concentration did not modify the associations between the serum n–6 PUFA concentrations and either QTc or JTc (*P*-interaction > 0.063).

## Discussion

In the current study of 1420 middle-aged and older males without CVD from eastern Finland, higher serum concentrations of total n–6 PUFA and LA were associated with lower QTc and JTc intervals. To our knowledge, the current study is the first to evaluate the associations of serum n–6 PUFA concentrations with QTc and JTc in humans. However, in a study on mice injected with dobutamine to simulate an exercise-induced stress test, a high intake of safflower oil—a source of n–6 PUFA—decreased the QT interval in both young and old mice [26]. In another animal study, the group injected with intravenous AA showed higher QT intervals compared with the control group [27]. In contrast, in our study, higher serum AA concentration was not associated with QTc and JTc.

A potential mechanism by which LA shortens the QTc interval may involve its effect on cardiac ion channels. LA appears to increase potassium channel activation and slow down deactivation, which impacts the repolarization phase of the cardiac cycle [28]. As a result, potassium channels open more rapidly and remain open for a longer duration, allowing the heart’s electrical activity to repolarize faster, leading to a shortened QT interval. In addition, modulation of calcium channels that affect cardiac dynamics may contribute to the shortening of the QT and JT intervals [29]. Furthermore, the anti-inflammatory effect of particularly LA may be another potential mechanism explaining the inverse association of LA with QT and JT intervals [30,31]. However, in our study, this is not a likely explanation because adjustment for the serum CRP had no impact on the associations with LA. Similar lack of an impact on the associations after adjustment for the serum lipids also suggested that the associations with QTc and JTc are not mediated by the effects of LA on the serum LDL or HDL cholesterol or triglycerides.

Our study had multiple strengths. It involved a large sample size and used data from an exercise test with ECG readings. Furthermore, the extensive KIHD database allowed for the consideration of many potential confounding variables. Moreover, instead of relying on dietary intake reports, we measured serum n–6 PUFA concentrations, reducing potential biases associated with misreporting and misclassification. In the KIHD cohort, both serum LA and AA concentrations associate with their dietary intakes. Finally, correcting QT and JT intervals for heart rate and excluding males with prolonged QRS complexes reduced interindividual variability, thereby enhancing the sensitivity of associations between the n–6 PUFA concentrations and ECG parameters.

A limitation of this study was the focus on middle-aged and older males from eastern Finland, which may limit the applicability of our findings to other demographics, including females and younger age groups. In addition, the cross-sectional design prevents us from establishing causal links between serum n–6 PUFA concentrations and ECG parameters. Unlike n–6 PUFA concentrations in adipose tissue and erythrocytes, which reflect long-term intake, serum fatty acids primarily indicate more recent intake [35] . Nevertheless, the baseline serum n–6 PUFA concentrations in our cohort were reasonably consistent with repeated measurements over several years (correlation coefficients ≥ 0.5) [32], indicating that they likely represent typical concentrations.

In conclusion, higher serum concentrations of total n–6 PUFA and LA were associated with shorter QTc and JTc intervals in middle-aged and older males. No significant associations were observed between serum concentrations of GLA, DGLA, or AA and QTc or JTc intervals. In addition to the known beneficial effects of LA, especially not only on serum lipids but also on glucose homeostasis and possibly also on low-grade inflammation [31,33,34], our finding may explain a potential mechanism of particularly LA in reduction of CVD risk. More large-scale studies in diverse populations across different age groups and sexes would be needed to confirm these findings. These studies could help in clarifying better the mechanisms through which n–6 PUFAs may influence cardiac electrophysiology.

## Author contributions

The authors’ responsibilities were as follows – HE, BT, T-PT, JKV: designed the research; T-PT, JTS, JK, SK, JKV: acquired data; HE, BT, JKV: statistically analyzed the data; JKV: had primary responsibility for final content; HE: drafted the manuscript; BT, T-PT, JK, JTS, SK, JKV: critically revised the manuscript for important intellectual content; and all authors: read and approved the final manuscript.

## Funding

This study was supported by the Orion Research Foundation to HE. The KIHD study was for the most parts funded by research grants from the Research Council of Finland to JTS. The funders had no role in the design, implementation, analysis or interpretation of the data.

## Conflict of interest

JTS is the CEO of MAS-Metabolic Analytical Services Oy. The other authors report no conflict of interest.
