# Supplementary material for: Associations of Serum n–6 Polyunsaturated Fatty Acid Concentrations with Heart Rate-Corrected QT and JT Intervals in Middle-Aged Males
Source: J Nutr. 2025 Sep 25;155(11):4016–22. doi: 10.1016/j.tjnut.2025.09.012 (PMC12799411; doi:10.1016/j.tjnut.2025.09.012)
Supplement: Multimedia component 1 [file mmc1.docx]

**Associations of serum n-6 polyunsaturated fatty acid concentrations with heart rate-corrected QT and JT intervals** **in middle-aged males**

Haleh Esmaili, et al.


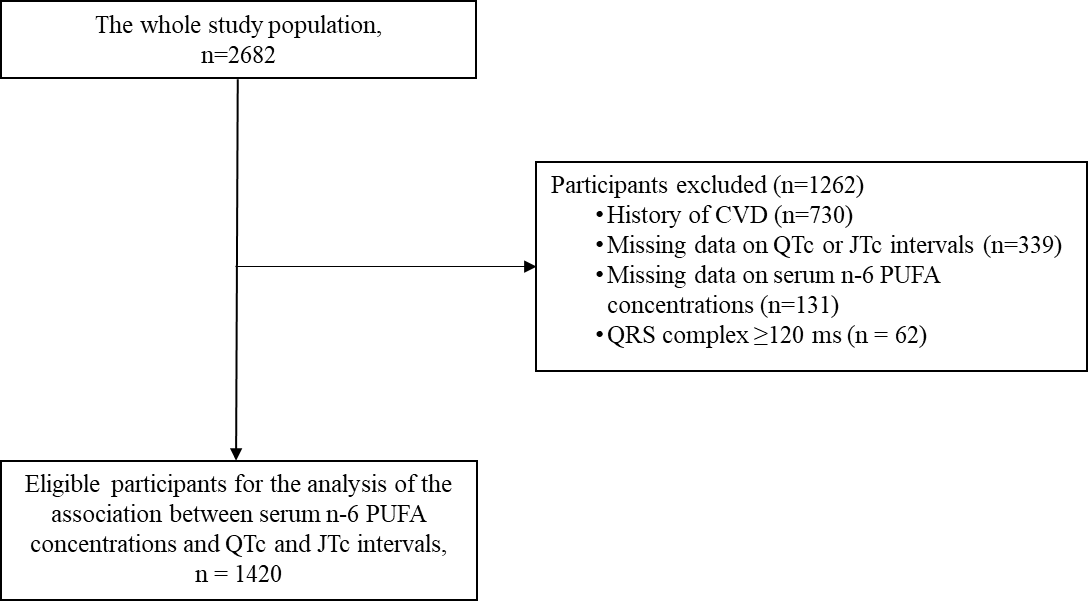


**Supplementary Figure 1.**

Participant flow-chart. CVD, cardiovascular diseases; PUFA, polyunsaturated fatty acids; QTc, heart rate-corrected QT interval; JTc, heart-rate-corrected JT interval.


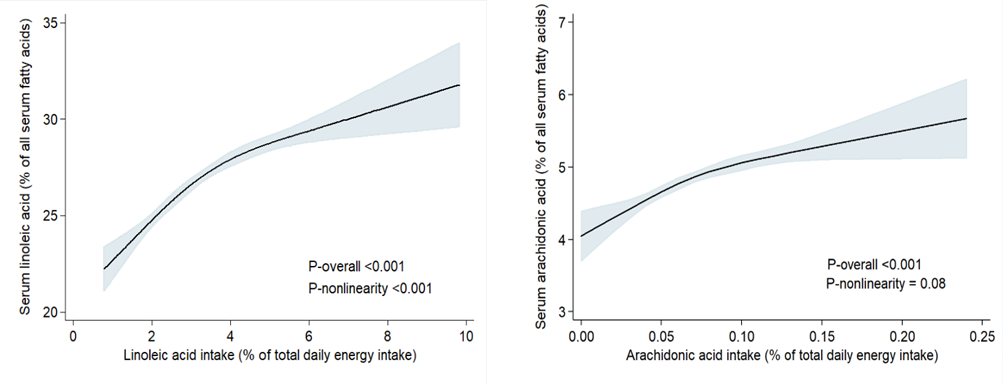


**Supplementary Figure 2.**

Associations of dietary linoleic acid and arachidonic acid intakes with their serum concentrations, evaluated using restricted cubic splines. The solid lines represent the central estimates and the shaded area the 95% confidence intervals. Median intake of linoleic acid was 3.1 percent of energy (8.3 g/day) and of arachidonic acid 0.06 percent of energy (0.17 g/day).
